# Development and Evaluation of a Novel Loop-Mediated Isothermal Amplification Assay for Diagnosis of Cutaneous and Visceral Leishmaniasis

**DOI:** 10.1128/JCM.00386-18

**Published:** 2018-06-25

**Authors:** Emily Rebecca Adams, Gerard Schoone, Inge Versteeg, Maria Adelaida Gomez, Ermias Diro, Yasuyoshi Mori, Desiree Perlee, Tim Downing, Nancy Saravia, Ashenafi Assaye, Asrat Hailu, Audrey Albertini, Joseph Mathu Ndung'u, Henk Schallig

**Affiliations:** a Research Centre for Drugs and Diagnostics, Parasitology Department, Liverpool School of Tropical Medicine, Liverpool, United Kingdom; b Academic Medical Centre, Department of Medical Microbiology, Parasitology Unit, Amsterdam, The Netherlands; c Centro Internacional de Entrenamiento e Investigaciones Médicas, CIDEIM, Cali, Colombia; d University of Gondar, Gondar, Ethiopia; e Eiken Chemical Company, Tokyo, Japan; f Parasite Genomics, Wellcome Trust Sanger Institute, Wellcome Trust Genome Campus, Hinxton, United Kingdom; g University of Addis Ababa, School of Medicine, Addis Ababa, Ethiopia; h Foundation for Innovative New Diagnostics, Geneva, Switzerland; i Universidad Icesi, Cali, Colombia; University of Texas Medical Branch

**Keywords:** LAMP, Leishmania, cutaneous, diagnostics, visceral

## Abstract

A novel pan-Leishmania loop-mediated isothermal amplification (LAMP) assay for the diagnosis of cutaneous and visceral leishmaniasis (CL and VL) that can be used in near-patient settings was developed. Primers were designed based on the 18S ribosomal DNA (rDNA) and the conserved region of minicircle kinetoplast DNA (kDNA), selected on the basis of high copy number. LAMP assays were evaluated for CL diagnosis in a prospective cohort trial of 105 patients in southwest Colombia. Lesion swab samples from CL suspects were collected and were tested using the LAMP assay, and the results were compared to those of a composite reference of microscopy and/or culture in order to calculate diagnostic accuracy. LAMP assays were tested on samples (including whole blood, peripheral blood mononuclear cells, and buffy coat) from 50 suspected VL patients from Ethiopia. Diagnostic accuracy was calculated against a reference standard of microscopy of splenic or bone marrow aspirates. To calculate analytical specificity, 100 clinical samples and isolates from fever-causing pathogens, including malaria parasites, arboviruses, and bacteria, were tested. We found that the LAMP assay had a sensitivity of 95% (95% confidence interval [CI], 87.2% to 98.5%) and a specificity of 86% (95% CI, 67.3% to 95.9%) for the diagnosis of CL. With VL suspects, the sensitivity of the LAMP assay was 92% (95% CI, 74.9% to 99.1%) and its specificity was 100% (95% CI, 85.8% to 100%) in whole blood. For CL, the LAMP assay is a sensitive tool for diagnosis and requires less equipment, time, and expertise than alternative CL diagnostics. For VL, the LAMP assay using a minimally invasive sample is more sensitive than the gold standard. Analytical specificity was 100%.

## INTRODUCTION

Infection with Leishmania parasites causes a spectrum of diseases, from self-healing skin ulcers—cutaneous leishmaniasis (CL)—to the potentially fatal form, visceral leishmaniasis (VL), which affects internal organs, in particular the spleen, liver, bone marrow, and lymph nodes. Accurate and opportune laboratory diagnosis followed by appropriate treatment is crucial for patient management and for decreasing transmission (1). Parasitological confirmation by microscopy of biopsy specimens, sometimes in combination with culture techniques, remains the reference standard of laboratory diagnosis for both VL and CL. For VL, serological diagnostics are available in the form of rapid diagnostic tests (RDTs) and enzyme-linked immunosorbent assays (ELISAs), optimally based on the rK39 antigen (2, 3), the direct agglutination test (DAT) (4), and immunofluorescent antibody tests (IFA/IFAT). However, due to poor specificity, these tests are recommended for use only after prolonged fever, which is most commonly taken to mean >14 days of fever (3). No alternative serological diagnostic test exists for CL, due to low concentrations of circulating antibodies; with regard to molecular tools, several diagnostic protocols have been validated for CL, but no reference standards are currently available.

Reference standard diagnostic tests suffer from challenges. Microscopy can be poorly sensitive, can lack standardization of tissue collection, and requires quality control and invasive sample types. The rK39 RDTs lack specificity and also sensitivity in certain regions, and culture methods require time, expertise, and considerable infrastructure.

A recent advance in nucleic-acid-based diagnostics has been the development of loop-mediated isothermal amplification (LAMP) of DNA. LAMP diagnostic kits have been developed for a variety of infectious diseases, including tuberculosis (5, 6), human African trypanosomiasis (HAT) (7), and malaria (8, 9). This nucleic acid amplification technique (NAAT) uses only one enzyme (*Bst* DNA polymerase) and is able to amplify large amounts of DNA within 40 min through the intricate design of primers and auto-cycling strand displacement DNA synthesis. A thermocycler is not required, because the enzyme works under isothermal conditions, and reagents are dried down with no requirement for a cold chain. Results can be read visually, and there is no postamplification handling or processing. This makes LAMP a powerful diagnostic test for settings where disease is endemic, bringing molecular diagnostics closer to the patient.

A LAMP assay for VL could specifically identify patients with this disease earlier than is currently possible, thus enabling earlier treatment. Recent modeling data for VL diagnostics show that early diagnosis and treatment of patients have the potential to greatly reduce the transmission of disease in elimination zones (1).

Attempts to develop a LAMP test for leishmaniasis have been made previously (10, 11). However, these were in-house assays rather than diagnostic kits and should not be considered for quality-controlled, standardized use. In addition, the design of some LAMP primer sets is geographically focused and therefore is not suitable for all areas of Leishmania endemicity (11). Here we describe the development of a novel LAMP assay, with the advantages of quality control and standardization that come with product development. We also present data collected during the development of the assay from cohorts of suspected CL patients in Colombia and suspected VL patients from Ethiopia.

## MATERIALS AND METHODS

### Target selection.

A combination of literature searches and sequence alignment identified potential target genes conserved within the genus Leishmania that had low homology (<80%) to other targets, including the DNA of Trypanosoma, the pathogen taxonomically nearest to Leishmania, and human DNA. The genus Leishmania was represented by the five reference genomes: Leishmania major MHOM/IL/1981/Friedlin (12), L. braziliensis MHOM/BR/1975/M2904 (13), L. mexicana MHOM/GT/2001/U1103cl25 (14), L. donovani MHOM/NP/2003/BPK282/0cl4 (15), and L. infantum (JPCM5) MCAN/ES/1998/LLM-87 (13). To exclude targets that could be amplified due to trypanosomiasis, the genus Trypanosoma was represented by Trypanosoma cruzi VI CL Brener (16), T. cruzi I Sylvio X10/1 (17), *T. vivax* Y486 (18), T. brucei TREU927 (19), and T. congolense IL-3000 (20). Sequence conservation and suitability as a LAMP target were assessed using genome-wide sequence alignment with MAVID (21) following construction of an orthology map with Mercator (22), which identified coding DNA sequence (CDS) regions with Genscan and measured orthology with BLAT and MUMmer. Sequences totaling 21 Mb in length were present in all five Leishmania species (average sequence length, ∼32 Mb) and absent in all five Trypanosoma species. A total of 14,030 candidate LAMP targets of >200 bp were identified; these spanned 7,942 kb of sequence. Gene length, GC content, and *in silico* copy number were evaluated using read information with SAMtools v0.1.11 (23), and a priority list of targets for primer design was made.

Further sequence alignment showed only 18 substitutions at the 2,190-bp 18S ribosomal DNA (rDNA) gene using GenBank PopSet 254847845 (24) across nine Leishmania species (the five species mentioned above plus L. amazonensis, L. guyanensis, L. panamensis, and L. tropica). The 3′ end (bp 1954 to 2190) of the 18S rDNA gene showed some homology to that of the Trypanosoma 18S rDNA gene.

### Copy number calculation.

In order to rank genes in order of priority for LAMP primer design, the copy number of each potential target was calculated experimentally as follows. Promastigotes were cultivated in RPMI 1640 culture medium containing 10% fetal calf serum at 27°C. DNAs from eight Leishmania species were extracted using the DNeasy extraction kit (Qiagen) at the log phase. For the purposes of this study, we refer to VL-causing species (L. donovani and L. infantum) and CL-causing species (L. tropica, L. major, L. braziliensis, L. mexicana, L. panamensis, L. guyanensis). Alignments were made using T-Coffee software (tcoffee.crg.cat) for all target genes except the kinetoplast DNA (kDNA), for which existing primer sets from the work of de Paiva Cavalcanti et al. (25) were used. Each target gene was amplified with HotStar *Taq* polymerase using the following protocol: 94°C for 10 min, followed by 40 cycles of 94°C for 30 s, either 52°C, 55°C, or 58°C for 30 s, and 72°C for 30 s, with a final hold of 72°C for 10 min. The resultant fragments were cloned as single-copy vectors using the TOPO TA cloning kit (Invitrogen). Colony PCR was used to select colonies containing the insert, and transformants were cultured in LB medium containing ampicillin (50 μg/ml). DNA was extracted using the Qiagen Plasmid Midi kit, and DNA digestion was performed for 2 h at 37°C with the EcoRI and HindIII/Xba restriction enzymes. Sybr green quantitative PCR (qPCR) was performed for each target gene with the following protocol: 95°C for 5 min, followed by 40 cycles of 95°C for 10 s and 60°C for 40 s. A 10-fold dilution series of vector DNA was used as a standard curve, where the vector was known to contain a single copy of the target gene and the weight of the vector was known. Additionally, a 5-fold dilution series of promastigote DNA was included, whereby the weight of the whole genome was known (14). The DNA concentrations of the vector and genomic DNAs were determined using a Thermo Scientific NanoDrop 1000 spectrophotometer. The cycle threshold (*C_T_*) of genomic DNA was compared with the *C_T_* of vector DNA, and the copy number was calculated by the standard formula, as [(known concentration of vector DNA)/(vector weight)]/[(concentration of genomic DNA calculated by qPCR)/(genome weight)], where concentrations are expressed in nanograms per microliter and weight is expressed in nanograms. The 7SL gene, a known single-copy-number gene (26), was used as a control.

### Primer design.

The 18S rDNA gene, histone H3 gene, and kDNA (Table 1) were chosen for primer design on the basis of their high copy numbers. PrimerExplorer software v4.0 (https://primerexplorer.jp/elamp4.0.0/index.html) was used to design LAMP primers on all three targets. Targets with the highest copy numbers were multiplexed to optimize sensitivity.

**TABLE 1:** Three highest-copy-number targets with low homology to related pathogens

Target	Copy no.	
	From the literature	Exptl
Kinetoplast DNA (visceral leishmaniasis)	>10,000	7,500–22,500
18S ribosomal DNA	20–200	300–2,200
Histone H3		80–380
7SL RNA	1	1

### LOD.

In order to determine the limit of detection (LOD) of each of the prototype LAMP primer sets, serial dilutions of DNA from eight species (see above) from different geographical areas, including Asia and Africa, were tested; these included four VL-causing strains and two CL-causing strains of the species L. infantum and L. donovani. Cultured promastigotes of Leishmania species were prepared, and DNA was extracted using the phenol-chloroform extraction method. A 10-fold dilution series was tested from 1,000 parasites per μl to 0.001 parasite/μl. To ensure that there was no cross-reactivity of the LAMP primer sets, they were tested with serial dilutions of DNA from Trypanosoma brucei, T. cruzi, Plasmodium falciparum, the human cell lines THP1 and U937, salmon sperm, human whole blood, and buffy coat.

### Bank of pathogen samples.

In order to ensure the specificity of the LAMP assay, 50 clinical samples or cultured isolates were tested. These included high-, medium-, and low-concentration samples of Plasmodium falciparum, P. vivax, Trypanosome brucei brucei, Trypanosoma cruzi, Giardia lamblia, and Cryptosporidium parvum, as well as high-, medium-, and low-concentration samples of 50 isolates of dengue virus, chikungunya virus, Zika virus, and bacterial species including Escherichia coli and Klebsiella pneumoniae.

### Cutaneous leishmaniasis clinical samples.

A prospective collection of samples from suspected CL patients from CIDEIM, Cali, Colombia (27), was used to estimate the diagnostic sensitivity of the developed prototype multiplex LAMP kit (kDNA plus 18S rDNA primers). One lesion swab sample (Isohelix DNA buccal swabs; SK-1S) was taken per suspected patient by gently rubbing a swab over the ulcer ∼10 times, and the sample was then stored at −20°C. A DNeasy blood and tissue kit (Qiagen, USA) was used to extract DNA according to the manufacturer's instructions, and the DNA was eluted in 50 μl distilled water. The diagnostic performance of LAMP was compared with a composite reference standard of microscopy and/or culture positivity. Briefly, two slides with three lesion smears on each slide were microscopically examined for amastigotes; parasite isolation in semisolid culture medium was attempted from 4 independent lesion aspirates from each participant. Parasite isolation was traced for a maximum of 1 month postinoculation.

### CL study ethics.

This study was approved and monitored by the institutional review board of the Centro Internacional de Entrenamiento e Investigaciones Médicas for ethical conduct of research involving human subjects.

### Visceral leishmaniasis clinical samples.

Blood was collected from 50 suspected VL patients from the University of Gondar Hospital, Amhara Regional State, Northern Ethiopia, in 2013. In order to determine the optimal extraction method with respect to parasite numbers, we compared the isolation of peripheral blood mononuclear cells (PBMC) with that of buffy coat and with that of whole blood stored in heparin tubes from the same patient samples. PBMC were isolated by slowly layering 2 ml heparin blood on top of an equal volume of Histopaque (Sigma-Aldrich), and the sample was centrifuged for 30 min at 2,000 rpm. The PBMC fraction was removed, suspended in 600 μl phosphate-buffered saline (PBS), and once again centrifuged for 1 min at 8,000 rpm. The cells were resuspended in 180 μl PBS. The buffy coat fraction was isolated by centrifuging 2 ml heparin blood for 5 min at 8,000 rpm. The buffy coat fraction was removed and was suspended in PBS to a total volume of 180 μl. All PBMC, buffy coat, and plain heparin blood samples (180-μl volumes) were mixed with an equal volume of AS1 buffer (Qiagen). DNA was extracted with a Qiagen Mini blood and tissue extraction kit according to protocol, and the resultant samples were stored at −20°C. LAMP and qPCR were compared to microscopy of either bone marrow aspirate or spleen aspirate as the gold standard.

### VL study ethics.

This study was approved and monitored by the University of Gondar Institutional Review Board, Gondar, Ethiopia (reference number R/C/S/V/P/05/664/2013). Written informed consent was obtained from patients for the use of their specimens in the study.

### Molecular methods. (i) LAMP.

LAMP (Eiken Chemical, Japan) was performed as per the manufacturer's instruction. A 3-μl volume of extracted DNA was added to a LAMP tube plus 27 μl water. Tubes were turned upside down for 2 min to release the dried-down reagents in the cap of the tube. Samples were briefly centrifuged and were then placed in a real-time turbidimeter (Eiken), first at 65°C for 40 min and then at 80°C for 2 min. A LAMP reaction was considered positive for Leishmania if fluorescence was observed visually and if a positive reaction was observed in the turbidimeter. Discrepancies between these two methods were recorded.

### (ii) qPCR.

qPCR was performed on DNA extracted from swab samples as described by Adams et al. in 2014, based on amplification of the 18S rDNA gene for CL diagnosis (27). kDNA qPCR was performed on all VL patient samples (*n* = 50). A 1.2-μl volume of DNA was added to 11.3 μl amplification mixture containing 6.25 μl iQ Supermix (catalog no. 170-8862; Bio-Rad), 0.25 μM kDNA forward primer (5′-TCCCAAACTTTTCTGGTCCT-3′), 0.25 μM kDNA reverse primer (5′-TTACACCAACCCCCAGTTTC-3′), and 0.12 μM kDNA probe (5′-6-carboxyfluorescein [FAM]-TTCTGCGAAAACCGAAAAATGGGTGC-BHQ-3′). The qPCR protocol (CFX-96; Bio-Rad) was as follows: 5 min at 95°C, followed by 40 cycles of 10 s at 95°C and 40 s at 54°C.

qPCR was used in this program as a comparator molecular method to LAMP to enable full understanding of results. The diagnostic accuracy of data, including sensitivity and specificity, was calculated for LAMP and qPCR. This study followed the STARD (Standards for Reporting of Diagnostic Accuracy Studies) guidelines, including blinding of index and reference diagnostic tests.

## RESULTS

### Target selection.

The relative copy numbers of nine different target genes were calculated for the eight Leishmania species tested, with averages across two strains. The results for the highest-copy-number targets are shown in Table 1, relative to the known single-copy target of the 7SL gene.

### Prototype primer sets.

Primer design was attempted on three target genes: the 18S rDNA gene and the histone H3 gene for pan-Leishmania assays and kDNA for a VL-specific assay. Each primer set was tested for the limit of detection (LOD) using a real-time LAMP turbidimeter. kDNA had the lowest LOD, at 0.0001 parasite/μl, on L. donovani and L. infantum and no amplification with CL-causing Leishmania species. The 18S rDNA gene had the next lowest LOD, at 0.01 to 0.001 parasite/μl. The histone LAMP primers had a LOD similar to that of the 18S rDNA gene at 0.01 parasite/μl but could not amplify all L. guyanensis and L. braziliensis strains, indicating low sequence homology to some South American strains. The 18S rDNA and kDNA targets were multiplexed to optimize sensitivity for detecting VL and the ability to detect all Leishmania species that cause CL; LOD was not affected by multiplexing. All testing of primer sets was performed using the dried-down LAMP assay developed by Eiken. No cross-reaction was observed with serial dilutions of DNA from T. brucei, T. cruzi, P. falciparum, salmon sperm, the human cell lines THP1 and U937, human whole blood, or buffy coat.

### Bank of pathogen samples.

Of 100 clinical samples and cultured isolates from different pathogens, none were positive with the Leishmania LAMP kit, which thus showed an analytical specificity of 100%.

### Cutaneous leishmaniasis samples.

A total of 105 clinical suspects were enrolled in the CL diagnostic study. A complete description of the demographic and clinical characteristics of the study participants and Leishmania species has been reported by Adams et al. (27). Parasites were isolated and identified for 64% of the participants; L. panamensis predominated overall, with representation from the Viannia and Leishmania subgenera. Compared to the reference standard of microscopy and/or culture, LAMP (kDNA plus 18S rDNA) was 95% sensitive (95% confidence interval [CI], 87.22% to 98.53%) and 86% specific (95% CI, 67.32% to 95.88%). This compared well with qPCR data on the same samples, which showed a sensitivity of 97% (95% CI, 91% to 100%) and a specificity of 84% (95% CI, 64% to 95%).

### Visceral leishmaniasis samples.

Fifty VL suspects were enrolled, of whom 26 were positive for VL by microscopy of splenic (*n* = 19) or bone marrow (*n* = 7) aspirates. Of the 26 VL-positive individuals, 27% (*n* = 7) were also positive for HIV.

The parasite load was quantified by qPCR in order to compare the extraction efficiencies of the different sample types (whole blood, buffy coat, and PBMC). Among the 26 individuals parasitologically positive for VL, the highest parasite load, as determined by the *C_T_* on kDNA qPCR, was found in the whole blood (*n* = 19), followed by buffy coat (*n* = 5) and PBMC (*n* = 1), of 25 qPCR-positive individuals.

For VL suspects from Ethiopia, the sensitivity of LAMP with whole blood was 92% (95% CI, 74.9 to 99.1%), and its specificity was 100% (95% CI, 85.8 to 100%). The sensitivity of kDNA qPCR was 96% (95% CI, 80.1 to 99.9%), and its specificity was 92% (95% CI, 73 to 99%). The sensitivities of the two tests were the same on buffy coat samples as on whole-blood samples, but that of LAMP decreased to 89% in PBMC samples (Table 2). LAMP specificity was highest with whole blood, at 100%, but dropped to 96% with other sample types (Table 2). qPCR had a specificity between 92% and 95% depending on the sample type. However, sample numbers are limited, and confidence intervals overlap. Notably, for all HIV-positive patients (*n* = 7), all sample types were positive by both qPCR and LAMP.

**TABLE 2:** Correlation between results of microscopy, PCR, and the LAMP assay^*a*^ on blood, PBMCs, and buffy coat from 50 suspected VL patients from Ethiopia

Sample and test	No. of results^*b*^ that were:				Sensitivity (%) (95% CI)	Specificity (%) (95% CI)
	TP	FN	TN	FP		
Whole blood						
PCR	25	1	22	2	96.1 (80.1–99.9)	91.7 (73–99)
LAMP	24	2	24	0	92.3 (74.9–99.1)	100 (85.8–100)
PBMC						
PCR	25	1	23	1	96.1 (80.1–99.9)	95.8 (78.9–99.9)
LAMP	23	3	23	1	88.5 (69.9–97.6)	95.8 (78.9–99.9)
Buffy coat						
PCR	25	1	23	1	96.1 (80.1–99.9)	95.8 (78.9–99.9)
LAMP	24	2	23	1	92.3 (74.9–99.1)	95.8 (78.9–99.9)

a Performed at the Gondar teaching hospital.

b TP, true positive; FN, false negative; TN, true negative; FP, false positive.

No discrepancies were reported between the visual analysis of the LAMP tubes for fluorescence and the real-time turbidimeter data.

## DISCUSSION

This study presents data on the development of a LAMP diagnostic kit capable of detecting both VL and CL. Target genes were chosen based on high copy number and conservation across multiple strains and species of Leishmania across geographic areas. 18S rDNA and kDNA primer sets were multiplexed to ensure a sensitive reaction for VL (kDNA) and the ability to detect all species causing CL (18S rDNA). Since the differential diagnosis and sample types of VL and CL do not overlap, the combination of primers is considered appropriate. Testing was performed on geographically distinct strains and species of Leishmania to ensure the production of a robust and reliable test. The multiplex LAMP was highly sensitive and specific, with a limit of detection between 0.01 to 0.001 parasite per μl for CL-causing species and 0.0001 parasite per μl for VL-causing species on purified DNA. LAMP was 100% specific when tested on a range of fever-causing organisms with epidemiology overlapping that of Leishmania; these included bacteria, malaria parasites, arboviruses, and other protozoans. LAMP was taken forward for testing on prospective clinical sample collections. In a cohort of suspected CL patients from southwest Colombia, LAMP was 95% sensitive (95% CI, 87.2% to 98.5%) and 86% specific (95% CI, 67.3% to 95.9%). In a study on 50 suspected VL patients, LAMP showed a sensitivity of 92.3% (95% CI, 74.9% to 99.1%) and a specificity of 100% (95% CI, 85.8% to 100%) on whole blood. Due to the reduced number of sample-handling steps and reduced *C_T_* values, the whole-blood sample type is preferable to PBMC and buffy coat for VL.

For CL samples, the LAMP test showed overlapping confidence intervals with qPCR and was more sensitive than culture and microscopy alone. The lower specificity of both qPCR and LAMP may represent patients not detected by the composite reference standard, since no perfect gold standard test for CL exists. Multiple studies, including those of Adams et al. (27) and Faber et al. (28), have concluded that the molecular tools are more sensitive for diagnosing CL, and therefore, individuals with false-positive results by molecular tools may be considered truly positive patients. Follow-up studies of participants need to be conducted in order to determine whether this is the case. Swab sampling on lesions was an appropriate collection method, although this would need adaptation to areas where nonulcerated lesions of CL are prevalent.

In VL samples, LAMP performed on DNA extracted from whole blood showed good sensitivity compared to microscopy of highly invasive biopsy samples. The kDNA qPCR was only slightly more sensitive (96%) than LAMP (92%) in the Ethiopian collection. The extraction of DNA from whole blood resulted in a higher parasite load in qPCR than the processing of either PBMC or buffy coat, in spite of the fact that, for the latter two methods, 10 times more blood was used for extraction (200 μl versus 2 ml). Using plain heparin blood for extraction is favorable, since a smaller volume of blood is required, and the processing of this sample type requires fewer handling steps. Microscopic examination on bone marrow or spleen aspirates will remain the gold standard, because parasite numbers are high in these sample types. However, based on these results, LAMP could potentially be used to confirm infection in the majority of patients, and then aspiration could be performed for those who are LAMP negative but remain VL suspects. This would circumvent aspiration for the majority of patients. Also, in areas where it is not possible to take biopsy samples due to a lack of appropriate medical facilities, LAMP may be used to confirm infection.

Modeling studies have suggested the use of highly specific diagnostic tools for the detection of VL in elimination zones (1). This would enable treatment of cases earlier than is currently possible in the diagnostic algorithm. Results for VL suspects in Ethiopia show a high specificity of 100% (95% CI, 85.8 to 100%), suggesting that LAMP may be a suitable tool for this purpose.

LAMP has been developed as a platform diagnostic tool and is now available for malaria, tuberculosis, and HAT, as well as a range of viral and bacterial infections. In this study, this powerful diagnostic tool has now been designed for leishmaniasis and has been tested on Leishmania suspects from Colombia and Ethiopia. LAMP is simple and does not require expensive equipment; therefore, it can be used in basic laboratory facilities with minimal DNA extraction facilities. Further development work and evaluation data are required. For CL, it would be useful to follow up patients with (false) positive LAMP reactions to see if they become positive with the gold standard. Since alternative diagnostics exist for VL, the use of LAMP may be to confirm suspected patients in areas with poor infrastructure, and before 14 days of fever have passed.
